# Topical levocabastine—a review of therapeutic efficacy compared with topical sodium cromoglycate and oral terfenadine on days with high pollen counts

**DOI:** 10.1155/S0962935195000809

**Published:** 1995-05

**Authors:** Marianela de Azevedo

**Affiliations:** 1 Serviço de Imunoalergologia Hospital de S. João Al Prof Hernâni Monteiro Porto 4200 Portugal

## Abstract

Levocabastine is a new H_1_-receptor antagonist specifically developed for the topical treatment of seasonal allergic rhinoconjunctivitis. Clinical experience to date clearly demonstrates that levocabastine eye drops and nasal spray are effective and well tolerated for the treatment of this allergic disorder. Analysis of data from a number of comparative trials reveals that topical levocabastine is at least as effective as sodium cromoglycate and the oral antihistamine terfenadine, even on days with high pollen counts (≥ 50 pollen particles/m^3^ ) when symptoms are severe. Coupled with a rapid onset of action and twice daily dosing, these findings make topical levocabastine an attractive alternative to other therapeutic approaches as a first-line therapy for the treatment, of this common condition.


Mediators of Inflammation 4, S21–S25 (1995)

### Topical levocabastine—a review of therapeutic efficacy compared with topical sodium cromoglycate and oral terfenadine on days with high pollen counts

Marianela de Azevedo

Serviço de Imunoalergologia, Hospital de S. João, Al Prof Hernâni Monteiro, 4200 Porto, Portugal

**LEVOCABASTINE is a new $H_1$-receptor antagonist specifically developed for the topical treatment of seasonal allergic rhinoconjunctivitis. Clinical experience to date clearly demonstrates that levocabastine eye drops and nasal spray are effective and well tolerated for the treatment of this allergic disorder. Analysis of data from a number of comparative trials reveals that topical levocabastine is at least as effective as sodium cromoglycate and the oral antihistamine terfenadine, even on days with high pollen counts ($\geq 50$ pollen particles/$m^3$) when symptoms are severe. Coupled with a rapid onset of action and twice daily dosing, these findings make topical levocabastine an attractive alternative to other therapeutic approaches as a first-line therapy for the treatment of this common condition.**

**Key words:** $H_1$-receptor antagonist, High pollen, Levocabastine, Seasonal allergic rhinoconjunctivitis, Topical antihistamine

### Introduction

Given the wide array of therapeutic agents available for the treatment of seasonal allergic rhinoconjunctivitis, including $H_1$-receptor antagonists, vasoconstrictors, topical corticosteroids and sodium cromoglycate, assessment of comparative efficacy is obviously of considerable importance for optimal patient management. Comparison of the true therapeutic efficacy of these different agents may be somewhat problematic. There are a number of reasons for this, most notably the placebo response or spontaneous improvement in symptoms observed following administration of any anti-allergic medication and particularly a topical drug. Response rates greater than 40% have been reported for placebo eyedrops and nasal sprays.[1] As the pollen count during the trial period may not always be sufficient for symptoms to develop fully, this placebo response may mask differences in therapeutic efficacy. A more realistic assessment of the comparative efficacy of different therapeutic approaches can be obtained by comparing efficacy on days with high pollen counts.

This review will focus on levocabastine, a new $H_1$-receptor antagonist, specifically developed for the topical treatment of seasonal allergic rhinoconjunctivitis and a comparison of the therapeutic efficacy of this agent with that of two other widely used and anti-allergic agents, the oral $H_1$-receptor antagonist terfenadine and the topical mast cell stabilizer sodium cromoglycate. In particular, emphasis will be placed on the comparative efficacy of these different therapeutic approaches for the treatment of seasonal allergic rhinoconjunctivitis on days with high pollen counts (defined as greater than or equal to 50 pollen particles/$m^3$).

### Levocabastine versus sodium cromoglycate:

A number of clinical trials have demonstrated that topical levocabastine is significantly more effective than sodium cromoglycate for the treatment of seasonal allergic rhinoconjunctivitis,[2–5] However, in only two of these trials were periods of high pollen counts sufficiently long to permit separate analysis of therapeutic efficacy as a function of the pollen count.[2,5]

Although these were independent trials, the study protocols were similar. Both were double-blind, parallel-group trials in patients with seasonal allergic conjunctivitis, with or without concurrent nasal symptoms. Patients were randomized to receive either levocabastine (0.5 mg/ml), sodium cromoglycate (20 mg/ml) or matching placebo eye drops at a dose of one drop in each eye four times daily for a period of 4 weeks. Both the patients and the investigators were required to provide global evaluations of therapeutic efficacy at the end of the trial. In addition, the investigators assessed a range of typical symptoms including ocular irritation, itching, redness, lacrimation and eyelid oedema, at the start of the trial, after 2 weeks of treatment and at the end of the study. Symptom severity

© 1995 Rapid Communications of Oxford Ltd

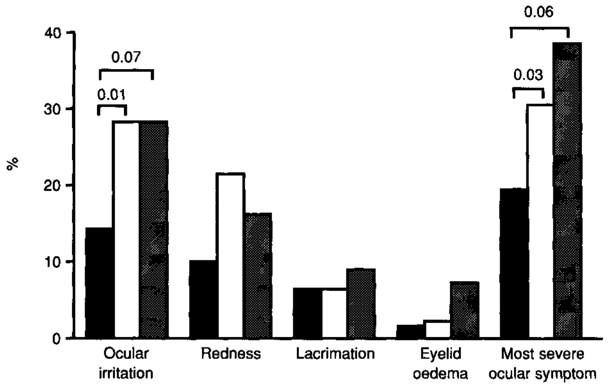

FIG. 1. Median area under the curve of daily symptom severity derived from the patients' diaries.[2]

was graded on a set scale where 0 = absent, 1 = mild, 2 = moderate, 3 = severe. The same symptoms were assessed by the patients on a daily basis and recorded on a visual analogue scale (VAS; 0 = absent, 100 = severe).

A total of 60 patients participated in the first study.[2] In all, 18 patients were randomized to receive levocabastine, 21 to receive sodium cromoglycate and 21 to receive placebo. After 4 weeks of treatment, the investigator rated global therapeutic efficacy to be excellent or good in 89% of levocabastine-treated patients compared with 67% of those who received sodium cromoglycate ($p$ = 0.03) and 48% of those in the placebo group ($p$ = 0.007).

Analysis of the patients' VAS ratings of symptom severity revealed a consistent trend in favour of levocabastine (Fig. 1). Statistically significant differences in favour of levocabastine were observed for the predominant symptom of ocular irritation and the most severe ocular symptom. The percentage of days when patients were free from all symptoms was 53% for levocabastine-treated patients compared with 31% for those treated with sodium cromoglycate ($p$ = 0.02) and 34% in the placebo treatment group ($p$ = 0.08).

This trend was maintained on days with high pollen counts (20% of the treatment period) (Table 1). Only 3% of levocabastine-treated patients experienced moderate or severe ocular symptoms on high-pollen days compared with 40% of cromoglycate-treated patients ($p$ = 0.01) and 36% of those who received placebo ($p$ = 0.008). For ocular irritation, the percentage of symptom-free high-pollen days was 57% in the levocabastine group compared with 28% in the sodium cromoglycate group ($p < 0.02$) and 25% in the placebo treatment group ($p < 0.03$). In addition, lacrimation was absent on 88% of high-pollen days in the levocabastine group compared with 64% ($p$ = 0.05) and 58% ($p$ = 0.01) of days in the other two treatment groups, respectively.

The incidence of adverse events was similar in all three treatment groups. Ocular irritation following application of the eye drops was the most frequently reported adverse reaction. This was reported by 13 patients in both the levocabastine and sodium cromoglycate treatment groups and eight of those treated with placebo.

These findings are supported by the results of another published study.[5] Twenty-eight patients received levocabastine eye drops, while 32 were treated with sodium cromoglycate and 29 received placebo. At the end of the 4-week treatment period, 87% of levocabastine-treated patients rated therapeutic efficacy as excellent or

**Table 1.** Pecentage of symptom-free days according to patients' diaries for the entire treatment period and on days with high pollen counts. Statistically significant intergroup differences are indicated (Kruskall-Wallis test, and if the Kruskall-Wallis test showed a significant difference amongst the three groups, the Mann-Whitney $U$-test was performed).[2]

| | Total period | | | High pollen days | | |
|---|---|---|---|---|---|---|
| Ocular irritation | | | | | | |
| Levocabastine | 63 | | | 57 | | |
| | | $p$ = 0.006 | | | $p < 0.02$ | |
| Cromoglycate | 36 | | $p < 0.06$ | 28 | | $p$ = 0.01 |
| | | $p$ = 0.44 | | | $p < 0.65$ | |
| Placebo | 44 | | | 25 | | |
| Most severe ocular symptom* | | | | | | |
| Levocabastine | 53 | | | 44 | | |
| | | $p < 0.02$ | | | | |
| Cromoglycate | 31 | | $p$ = 0.08 | 27 | | KW $p$ = 0.21** |
| | | $p$ = 0.85 | | | | |
| Placebo | 34 | | | 21 | | |

*Ocular irritation, redness, lacrimation or swollen eyelids.
**Kruskall-Wallis (KW) one-way analysis of variance.

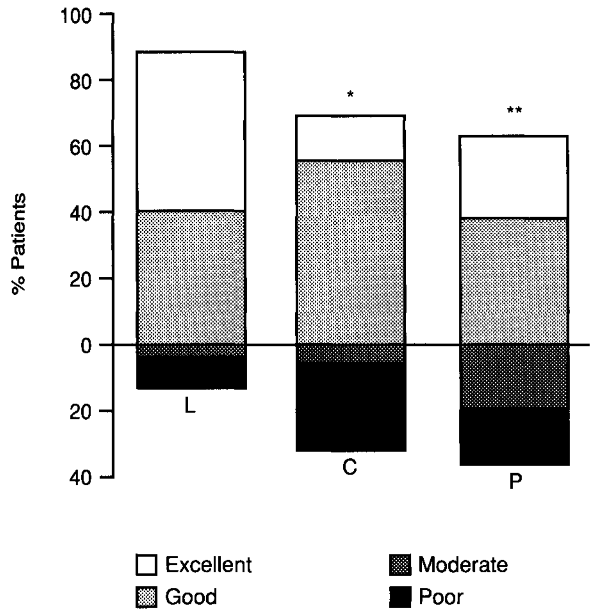

FIG. 2. Patients' global evaluations of therapeutic efficacy at the end of the trial. $^*p = 0.05$, $^{**}p = 0.006$ (Mann–Whitney *U*-test).[5] Reproduced with the kind permission of Munksgaard Int. Publishers Ltd, Copenhagen, Denmark.

good compared with 68% of those treated with sodium cromoglycate ($p = 0.006$) and 63% of the placebo treatment group ($p = 0.05$) (Fig. 2). Symptom severity was generally lower in the levocabastine treatment group. Investigator assessments revealed that levocabastine provided significantly greater relief of nasal symptoms after 2 weeks of treatment than either sodium cromoglycate ($p < 0.01$) or placebo ($p < 0.01$). This is of interest, as levocabastine was only administered ocularly and systemic absorption of levocabastine is reported to be minimal following

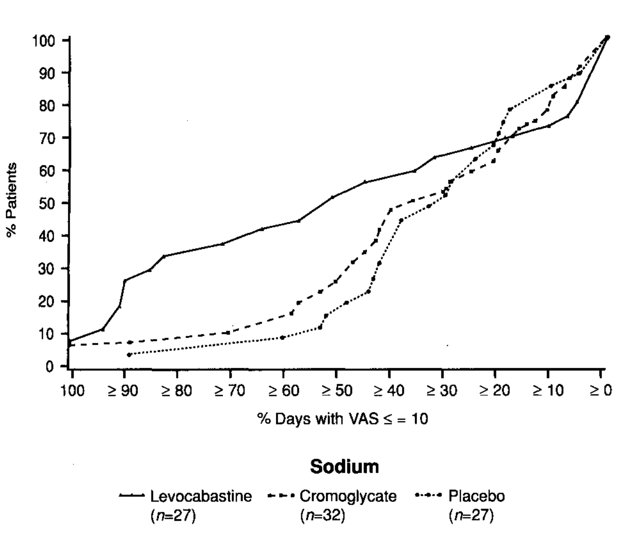

FIG. 3. Cumulative distributions of percentages of virtually symptom-free (VAS $\leqslant$ 10) high-pollen days in the three treatment groups.[5] Reproduced with the kind permission of Munksgaard Int. Publishers Ltd, Copenhagen, Denmark.

ocular administration.[1] Drainage of the levocabastine eye drops through the lacrimal ducts into the nasal passages is the most likely explanation for this effect.

After 4 weeks of treatment, lacrimation ($p < 0.01$), ocular redness ($p < 0.05$) and the most severe ocular symptom ($p < 0.05$) were significantly less severe in levocabastine-treated patients than in those who received sodium cromoglycate. Analysis of the patients' diaries revealed that 37% of patients in the levocabastine group were virtually symptom-free (VAS ratings $\leqslant$ 10) for at least 75% of the treatment period compared with only 6% of cromoglycate-treated patients ($p < 0.01$) and 4% of the placebo group ($p < 0.01$).

This trend was maintained on days with high pollen counts (approximately 54% of the study period) (Fig. 3). A total of 33% of levocabastine-treated patients were virtually symptom-free on high-pollen days compared with only 6% of those who received sodium cromoglycate ($p = 0.02$) and 4% of the placebo group ($p = 0.02$).

Both levocabastine and sodium cromoglycate were well tolerated. As expected, ocular irritation following administration of eye drops was the most frequently reported adverse effect with an incidence of 17.9% for levocabastine, 15.6% for sodium cromoglycate and 27.6% in the placebo treatment group.

### Levocabastine versus oral terfenadine

To date, three independent, randomized, double-blind, double-dummy, parallel-group trials have been published which assess the comparative efficacy of topical levocabastine and oral terfenadine for the treatment of seasonal allergic rhinoconjunctivitis.[6–8] Patients were randomized to receive either levocabastine eye drops (0.5 mg/ml, one drop in each eye twice daily) and nasal spray (0.5 mg/ml, two puffs in each nostril twice daily) plus a twice daily oral placebo or to receive oral terfenadine (60 mg twice daily) in combination with placebo eye drops and nasal spray for a total of 8 weeks.

Both the patients and the investigators performed a global evaluation of therapeutic efficacy at the end of the study period. In addition, the investigators rated the severity of ocular symptoms of redness, itching, lacrimation and eyelid oedema and nasal symptoms of sneezing, rhinorrhoea, itching and congestion on a scale from 0 to 3 (0 = absent, 1 = mild, 2 = moderate and 3 = severe) at the start of the trial and after 4 and 8 weeks of treatment. The patients were required to assess these symptoms on a daily basis using a VAS (0 = absent, 100 = severe).

The results of these studies show that levocabastine eye drops and nasal spray are at least as effective as oral terfenadine for the treatment of this allergic condition and statistically significant differences in favour of topical levocabastine were reported even though patients in the terfenadine group also benefited from the use of placebo eye drops and nasal spray. In particular, the available data suggest that topical levocabastine is more effective than oral terfenadine on days with high pollen counts.[6,8] A total of 115 patients with a documented history of grass and/or birch pollen-induced allergic rhinoconjunctivitis participated in the larger of these two trials,[8] 58 of whom were randomized to receive topical levocabastine. Both treatment regimens were well-tolerated and the incidence and type of adverse reactions were similar in the two treatment groups.

Global evaluations of therapeutic efficacy revealed a consistent, yet non-significant, trend in favour of the topical approach. However, after 4 weeks of treatment, investigator assessments revealed that the severity of ocular redness and the most severe ocular symptom were significantly lower in the levocabastine group than in the terfenadine group ($p < 0.01$ and $p < 0.05$, respectively). Analysis of the patients' diaries revealed that VAS ratings were significantly lower in the levocabastine group for ocular and nasal itching ($p < 0.05$), lacrimation ($p = 0.001$) and the most severe ocular symptom ($p < 0.05$). In addition, the percentage of symptom-free days was generally higher in the levocabastine group, while the percentage of days with severe symptoms tended to be lower.

High pollen counts were recorded during a consecutive period of 2 weeks. During this period, levocabastine was consistently more effective than oral terfenadine at controlling symptoms of seasonal allergic rhinoconjunctivitis. The incidence of severe lacrimation and ocular itching was significantly lower in the levocabastine group on days with high pollen counts ($p < 0.05$), while the percentage of days free from ocular and nasal itching ($p < 0.05$) and lacrimation ($p < 0.01$) was significantly higher (Fig. 4).

These findings are supported by those of a smaller trial initiated primarily to assess the tolerability of levocabastine eye drops.[6] In this study, 13 patients were randomized to receive topical levocabastine while 14 were treated with oral terfenadine. Use of oral medication and eye drops was mandatory, however, patients were requested only to use the nasal spray as required. The use of nasal spray was lower in the levocabastine group (46%) than in the terfenadine group (56%), suggesting that topical levocabastine was more effective at relieving nasal symptoms than oral terfenadine.

In all, 88% of levocabastine-treated patients considered the effect of treatment on ocular symptoms to be excellent or good compared with 75% of those who received terfenadine, while 75% of patients in each group were satisfied with the effect of the study medication on nasal symptoms. Investigator assessments revealed that symptom severity was consistently lower in the levocabastine treatment group. In particular, the severity of ocular itching was significantly lower ($p = 0.02$) in levocabastine-treated patients than in those who received terfenadine after 8 weeks of treatment.

Analysis of the patients' VAS ratings revealed that levocabastine was significantly more effective than terfenadine for sneezing ($p = 0.03$), rhino-

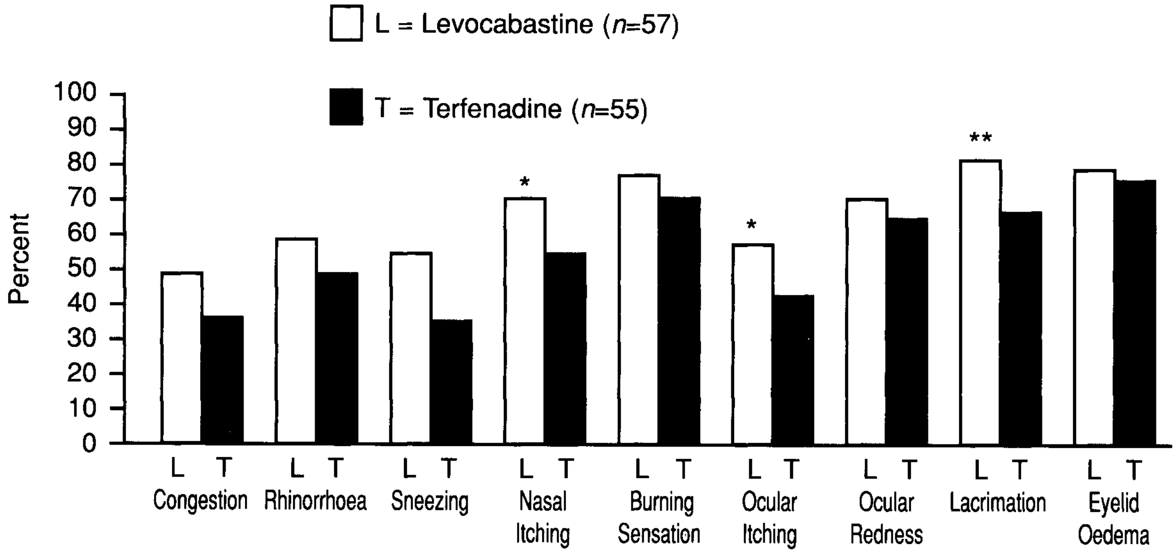

FIG. 4. Percentage of symptom-free high-pollen days in the two treatment groups. *$p \leqslant 0.05$, **$p \leqslant 0.01$.[8] Reproduced with the kind permission of Mosby Year Book Inc., St. Louis, MO, USA.

rrhoea ($p = 0.05$) and the most severe nasal symptom ($p = 0.02$). Furthermore, the percentage of days with severe nasal congestion ($p = 0.01$), rhinorrhoea, sneezing, itching, the most severe nasal symptom ($p < 0.01$) and the most severe of all symptoms ($p = 0.04$) were also significantly lower in the levocabastine group.

Analysis of therapeutic efficacy as a function of the pollen count revealed that this trend was maintained on high pollen days. Levocabastine provided significantly greater relief from all nasal symptoms ($p = 0.001–0.02$) and for the most severe of all symptoms ($p = 0.04$) than oral terfenadine on days when the pollen count was high.

Both treatment regimens were well tolerated. Ocular irritation was the most common adverse reaction with a similar incidence in the two treatment groups.

### Implications for patient management

Clinical experience to date clearly demonstrates that levocabastine eye drops and nasal spray are effective and well tolerated for the treatment of seasonal allergic rhinoconjunctivitis[9] with a number of comparative trials revealing that topical levocabastine is at least as effective as sodium cromoglycate and oral terfenadine for the treatment of this common condition, even on days with high pollen counts. Studies have shown that treatment efficacy is maintained for up to 4 months,[10] indicating that topical levocabastine is suitable for long-term therapy throughout the hay fever season.

Topical levocabastine has a number of distinct advantages over other agents used to treat seasonal allergic rhinoconjunctivitis. Firstly, levocabastine has an extremely rapid onset of action providing almost immediate relief from symptoms.[11,12] Moreover, unlike sodium cromoglycate, levocabastine is also effective when administered after allergen challenge.

In addition, the duration of action of levocabastine is sufficient to permit a convenient, twice-daily schedule.[13] Patient compliance with such a regimen is likely to be good. In contrast, other topical agents for the treatment of seasonal allergic rhinoconjunctivitis must be administered as frequently as six times daily.

It is obviously important that any anti-allergic medication is well tolerated during long-term therapy. Although oral $H_1$-receptor antagonists such as terfenadine are generally well tolerated, topical application of a $H_1$-receptor antagonist is preferable as a topical drug is associated with a minimal risk of systemic adverse effects. Levocabastine eye drops and nasal spray are both well tolerated. Local irritation following administration is the most frequently reported adverse reaction associated with topical levocabastine, however the incidence is comparable with that observed following administration of placebo or sodium cromoglycate.[1]

In conclusion, topical levocabastine is an attractive alternative to other common therapeutic approaches for the treatment of seasonal allergic rhinoconjunctivitis and the available clinical data clearly support its use as a first-line therapy for the treatment of this common condition.

### References

1. Dechant KL, Goa KL. Levocabastine. A review of its pharmacological properties and therapeutic potential as a topical antihistamine in allergic rhinitis and conjunctivitis. *Drugs* 1991; **41:** 202–224.
2. Azevedo M, Castel-Branco MG, Ferrez Oliveira J, *et al.* Double-blind comparison of levocabastine eye drops with sodium cromoglycate and placebo in the treatment of seasonal allergic conjunctivitis. *Clin Exp Allergy* 1991; **21:** 689–694.
3. Palma-Carlos AG, Chieira C, Conde TA, Robalo Cordeiro JA. Double-blind comparison of levocabastine nasal spray with sodium cromoglycate nasal spray in the treatment of seasonal allergic rhinitis. *Ann Allergy* 1991; **67:** 394–398.
4. Schata M, Jorde W, Richarz-Barthauer U. Levocabastine nasal spray better than sodium cromoglycate and placebo in the topical treatment of seasonal allergic rhinitis. *J Allergy Clin Immunol* 1991; **87:** 873–878.
5. Davies BH, Mullins J. Topical levocabastine is more effective than sodium cromoglycate for the prophylaxis and treatment of seasonal allergic conjunctivitis. *Allergy* 1993; **48:** 519–524.
6. Bahmer FA, Ruprecht KW. Safety and efficacy of topical levocabastine compared with oral terfenadine. *Ann Allergy* 1994; **72:** 429–434.
7. Rosenhall L, and the Livostin Study Group. A comparison of topical levocabastine and oral terfenadine in the treatment of allergic rhinoconjunctivitis. *Allergy* 1993; **48:** 530–534.
8. Søhoel P, Freng BA, Kramer J, *et al.* Topical levocabastine compared with oral terfenadine for the treatment of seasonal allergic rhinoconjunctivitis. *J Allergy Clin Immunol* 1993; **92:** 73–81.
9. Knight A. The role of levocabastine in the treatment of allergic rhinoconjunctivitis. *Br J Clin Pract* 1994; **48:** 139–143.
10. Frostad AB, Olsen AK. A comparison of topical levocabastine and sodium cromoglycate in the treatment of pollen-provoked allergic conjunctivitis. *Clin Exp Allergy* 1993; **23:** 406–409.
11. Palma-Carlos AG, Palma-Carlos ML, Rombaut N. The effect of levocabastine nasal spray in nasal provocation tests. *Int J Clin Pharmacol Res* 1988; **8:** 25–30.
12. Stokes TC, Feinberg G. Rapid onset of action of levocabastine eye drops in histamine-induced conjunctivitis. *Clin Exp Allergy* 1993; **23:** 791–794.
13. Tomiyama SA, Ohnishi M, Okuda M. The dose and duration of effect of levocabastine, a new topical $H_1$-antagonist, on nasal provocation reaction to allergen. *Am J Rhinology* 1993; **7:** 85–88.
